# Pushing through the Barriers: Peer Advice to Increase Physical Activity and Reduce Dementia Risk from Participants in a Massive Open Online Alzheimer’s Focused Course

**DOI:** 10.14283/jpad.2023.42

**Published:** 2023-04-11

**Authors:** M. R. Abela, H. Maxwell, A. Bindoff, J. Alty, M. Farrow, K. Lawler

**Affiliations:** 1 Wicking Dementia Research and Education Centre, College of Health and Medicine, University of Tasmania, Hobart, Tasmania 7001 Australia; 2 School of Health Sciences, University of Tasmania, Sydney, New South Wales Australia; 3 Neurology Department, Royal Hobart Hospital, Hobart, Tasmania Australia; 4 School of Allied Health, Human Services and Sport, La Trobe University, Melbourne, Victoria Australia

**Keywords:** Dementia, Alzheimer’s, disease, peer support, online learning, physical activity and ageing

## Abstract

**Background:**

Engagement in physical activity is associated with reduced dementia risk but insufficient physical activity is a global trend.

**Objectives:**

We aimed to explore what advice might be offered to others to increase physical activity and to identify enablers and barriers to physical activity for adults interested in dementia prevention and participating in a massive open online course.

**Participants:**

Two thousand, one hundred and thirty-two participants contributed to an online discussion forum.

**Design:**

Analysis was conducted using Topic modelling analysis followed by thematic analysis.

**Results:**

The themes generated from the discussion posts included time constraints, poor health and lack of motivation as barriers to physical activity, and social interaction, incidental activities, and dog ownership as enablers. Peer advice was frequently suggested around scheduling physical activity into the day and joining a friend or organised activity.

**Conclusion:**

This online discussion forum uniquely captured ideas from a large, diverse group of participants. Future research may benefit from further examining the role of discussion forums and peer advice in dementia risk reduction initiatives.

## Introduction

**P**hysical activity is recognised as a modifiable health behaviour for the prevention of cognitive decline and dementia including Alzheimer’s disease (1–4). Dementia is a progressive neurodegenerative condition characterised by decline in executive function, memory and attention. Further, it is associated with limitations in physical performance, declines in muscle strength, balance, and mobility, impacting on a person’s quality of life (5). With increasing life expectancies, dementia prevalence is rising. Around 50 million people live with dementia worldwide and this figure is projected to increase to 152 million by 2050 (6).

Physical activity has the potential to delay the onset of dementia and slow its progression and this could reduce dementia incidence (7, 8). Unfortunately, insufficient physical activity is a global trend (9). Researchers continue to monitor current levels and trends associated with insufficient physical activity. Whilst is has been reported that people have some knowledge of the benefits that physical activity can bring, this knowledge is generally at a level that is of a basic understanding, where people report’ it is good for health’ (10). Improving and closing this knowledge gap through health promotion initiatives may help to inform physical activity strategies, including those for dementia risk reduction.

Peer advice and support has been identified as an enabler of physical activity participation in a range of cohorts and may help address some cultural and social issues. For example, a systematic review of older people’s perspectives on physical activity participation found interaction with peers and encouragement from others were key social influences (11). Peer advice programs have also effectively encouraged people with heart disease to continue to be physically active after completing cardiac rehabilitation (12). These studies have generally considered social interaction and the development of social capital and support for physical activity from the perspective of face-to-face interactions. However, the COVID-19 pandemic has brought new challenges to the way individuals interact, accelerated the uptake of digital and online technologies, and resulted in extended periods of social isolation and inactivity for many across the globe (13). Safe and physically distanced peer support through online forums may contribute to helping the community regain and maintain adequate physical activity levels. Online forums can also be a rich source of qualitative data, that may offer additional insights compared to face-to-face interviews with researchers (14).

Understanding the barriers and enablers to physical activity participation for people wishing to reduce their risk of dementia will help inform targeted health promotion activities. Research commonly identifies affordability, accessibility, support of family and friends and a safe environment as enablers of participation in physical activity and ill-health, lack of time, caring responsibilities, costs, and poor environmental conditions as barriers (15, 16). Barriers and enablers to physical activity have been investigated across a range of cohorts including Indigenous Australians (17), Muslims (18), pregnant women (19) and older adults (16, 20). Barriers and enablers can differ between these groups, and successful physical activity initiatives may require bespoke accommodations such as changes to clothing requirements, culturally appropriate activity leaders, and considerations of age, gender, cultural expectations, and social responsibilities (18, 20, 21). Whilst there is ample information about barriers and enablers to physical activity participation, there has been little research specifically targeting these in relation to dementia prevention. It is possible that a strong desire to reduce the risk of developing dementia will influence barriers and enablers.

The aim of this study was to identify peer-advice about physical activity participation shared by people learning about dementia risk reduction, along with barriers to, enablers of, physical activity

## Methods

### Ethics Approval

This study was approved by the Tasmanian Social Sciences Human Research Ethics Committee (reference number H0015773) with all participants providing informed consent.

### The Preventing Dementia Massive Open Online Course (PD MOOC)

The PD MOOC is a free short course providing information about the potential for dementia prevention at a population level and risk reduction at an individual level. The course is presented by experts in dementia prevention research (22). Since its inception in 2016, it has attracted over 100,000 international participants with an average completion rate of 43%. The course is run twice per year and over 20,000 people enrol in each iteration. It is comprised of five modules released on a weekly basis, with each module consisting of approximately two hours of content and activities. Course materials range from written content to images, videos, discussions boards, quizzes, and animations. The five modules include, 1) can dementia be prevented? 2) Risk factors for dementia, 3) Dementia risk - it’s not all in your head, 4) A healthy and active mind and 5) Interventions for prevention (22).

### Participants

Participants were a convenience sample of people enrolled in the two 2019 iterations of the PD MOOC. The PD MOOC was actively marketed via a range of strategies including radio and print media interviews, paid print, online and social media advertising, and presentations and presence at community events. The PD MOOC was open to anyone with an interest in participating and the means to do so (internet access), anywhere in the world. On commencing the course, participants were presented with an online information sheet explaining the use of data they provided while undertaking the PD MOOC for research purposes and asked to provide consent by completing an online form. The only inclusion criterion was being 18 years of age or older.

### Data collection

In the second module of the PD MOOC, participants were given information about the link between regular physical activity and a reduced risk of dementia. They were invited to contribute to an optional discussion board, titled’ increasing our physical activity.’ The discussion board included the following introductory text.

“Participating in physical activity is not easy for everyone. This discussion board gives us an opportunity to talk about ways to get more active. We would love you to share your experiences and ideas with the group. Some starting points for conversation are:

1. Are there things that make it difficult for you to participate in more physical activity?
2. If you have increased your physical activity levels, what tips could you give others to help them do the same?

There are no right or wrong answers. Just tell us what you think or pose a question for your fellow MOOC participants”.

During the PD MOOC, members of the research team (KL, JA) also contributed comments to the board, to encourage participants to post their ideas, and to thank participants for their thoughts. Members of the research team did not provide advice about physical activity and commented so as not to reinforce or encourage a particular idea or thought, to seek the true views of the participants. Only the first post made by a participant was examined to gather participants’ initial thoughts. First posts were identified using system-generated user identification (ID) and timestamps.

Demographic data were extracted alongside the full content of the participants’ first posts. These included age, gender, highest level of education and employment organisation type, and were provided by participants via a course enrolment survey.

### Data analysis

Dual methods were used to analyse this large data set, informed by (23). Structural topic modelling, which uses a computer algorithm to identify topics in text data, enabled initial interrogation of the large set of text data (24) and Thematic Analysis (TA) informed by the methods of (25), then facilitated an in-depth exploration of the meaning of the data in relation to our phenomena of interest: barriers to physical activity, enablers of physical activity, and peer-advice about physical activity participation.

### Topic Modelling Analysis

Discussion forum text data were prepared before analysis by removing names to preserve privacy and anonymity. Words were tokenized by converting to lower case, removing punctuation and html tags, and stemming using the Porter algorithm (26). Stemming assigns a unique token to all words with the same semantic meaning (e.g. ‘run’ and ‘running’). No tokens were excluded except a unique token used to mask participant names. This means all stop-words (e.g., ‘and’, ‘the’, ‘they’) were retained as they may provide context (27).

Topic models were fitted by a researcher (AB) using methods as previously described (22, 23). Topics were identified within participant responses by analysing the co-occurrence and exclusivity of tokens using a probabilistic topic modelling approach. This approach relies on the assumption that frequently co-occurring words carry semantic meaning. A limitation of the algorithm is that the number of topics must be specified, so we fitted 9, 12, 15, and 18 topic models using the stm R package (28). In each model, the participant posts most representative of each topic, the words that most co-occurred within a topic, and the words that were most exclusive to each topic, were identified. Semantic coherence, term exclusivity, and exemplar relevance were assessed for each model and compared between models using the stm and stmQuality R packages (29).

A primary innovation of the ‘stm’ package is that it enables estimation (and comparison) of topic prevalence between groups by inclusion of covariates in the model. We included age, gender, and highest level of formal education as covariates.

### Secondary thematic analysis

Two researchers (MA, HM) familiarised themselves with the data by independently reviewing exemplars of the most representative participant posts for each topic in the 9, 12, 15 and 18 computer-generated topic models and made notes of any initial observations. This interrogation assured identification of coherent and meaningful patterns that could be used to determine major themes and examples of barriers, enablers and peer advice for physical activity participation for the MOOC cohort. These researchers then compared their findings and reached a strong consensus that the 15-topic model provided the most parsimonious and semantically coherent fit (23).

To understand the meaning of each topic, themes related to physical activity barriers, enablers and peer advice were developed and the researchers then reached consensus on the themes and the most appropriate data exemplars to illustrate them.

## Results

A total of 2132 participants in the 2019 iterations of the PD MOOC consented to research and contributed to the ‘increasing our physical activity’ discussion board. Table 1 shows the demographics of participants. The majority were female (87%) with 54% aged 55 years and over. Most participants were tertiary educated (67%) and reported working within health and aged care settings (43%).

**Table 1:** Participant demographics

**Age**	**n (%)**
< 25	100 (4.7)
25 – 34	179 (8.4)
35 – 44	210 (9.8)
45 – 54	414 (19.4)
55 – 64	689 (32.3)
65+	475 (22.3)
Missing	65 (3)
Gender	
Women	1853 (86.9)
Men	270 (12.7)
Prefer not to say	9 (0.4)
Employment Sector	
Community Health	422 (19.7)
Education	101 (4.7)
Government	44 (2.1)
Hospital	164 (7.7)
Non-Government Organisation	34 (1.6)
Residential Aged Care	332 (15.6)
Missing	824 (38.6)
Other	201 (9.9)
Education	
Higher University degree (Honours, Grad Dip, Masters, PhD)	523 (24.5)
Bachelor’s degree	552 (25.9)
Diploma or Associate degree	353 (16.6)
Certificate or Apprenticeship	222 (10.4)
High School	270 (12.7)
Primary School	6 (0.3)
Other	2 (0.1)
Missing	204 (9.6)
Total	2132

Note: Missing relates to participants not selecting an option on the survey

**Figure 1:**
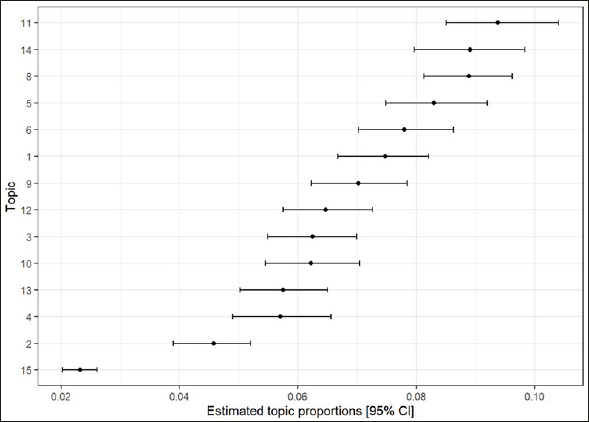
Estimated topic proportions for the fifteen topics

The fifteen topics identified by the structural topic model and thematic analysis are represented by data extracts. Figure 1 considers the topics from the perspective of the whole participant cohort. The themes are presented in the order determined by topic proportions over the entire data corpus identified by the TMA process There were some consistencies of terms in the participants responses and therefore there was some overlap in the themes across topics. This could possibly reflect the interrelatedness of the number of tenets to physical activity. Participants are identified by gender (F/M) and age (years). Following the TMA process, posts within each of the fifteen topics were thematically analysed resulting in contextualised interpretations (Table 2). Fourteen of the fifteen topics were interpreted. Topic 7 was excluded from further analysis due to the high frequency of third-party quotes in the representative sample.

**Table 2:** The 15-topic model generated by topic modelling analysis is illustrated with themes generated from the analysis of posts associated with each topic

**Structural topic modelling topics**	**Interpretation from thematic analysis**			
**Original Topic**	**Frequently co-occurring words**	**Most exclusive words**	**Theme identifier**	**Description**
1	enjoy, love, group, exercis, find, danc, class	love, danc, zumba, music, enjoy, someth, group	Enjoyment, organised activity and socialisation	Activities to enhance physical activity enjoyment
2	year, now, still, weight, life, bodi, diet	blood, diabet, lost, took, diet, sugar, told	modifying & finding physical activity relative to health issues	Health related issues and how physical activity improved health
3	exercis, get, week, realli, import, think, physic	inspir, thank, togeth, mum, support, import, realli	Understanding the benefits of physical activity	Knowledge of the benefits of physical activity for health
4	need, activ, exercis, physic, walk, like, make	need, answer, young, wear, lift, cloth, realis	Offerings of advice and support	Advice around physical activity
5	exercis, motiv, can, help, find, get, make	problem, knee, someon, pain, injuri, definit, arthriti	Ageing, fatigue and injury concerns; overcoming these concern	Barriers and enablers to physical activity participation
6	time, week, exercis, work, day, walk, find	time, manag, carer, three, min, school, mother	Work and family commitments	Time poor for physical activity
7	exercis, play, cycl, can, age, etc, year	bad, wish, cycl, game, real, pretti, play	Really need exercise, although it can be hard sometimes	Sacrifice and persistence-verbatim quotes dominate - exclude
8	Walk, dog, day, get, also, park, shop	Dog, park, shop, beach, away, walk, bush	Dog ownership, deliberate incidental activity	Opportunities for physical activity
9	exercis, well, physic, activ, health, class, year	well, notic, interact, improv, practic, joint, attend	Classes, programmes, mens shed	Programmes to engage in for physical activity
10	feel, walk, day, exercis, gym, start, activ	agre, cost, feel, rain, energet, minimum, fresh	Self-discipline and getting into routine	Being disciplined for physical activity
11	Work, day, activ, get, phys,exerc, time	work, job, tire, full, busi, kid	Work and family commitments	Time poor for physical activity
12	use, exercis, walk, bike, gym, now, train	use, instead, stair, transport, resist, bike, ride	Active transport	‘Active transport’ for physical activity opportunities
13	exercis, like, walk, just, thing, goodd jog	jog, couch, stick, housework, habit, recommend, afternoon	Technology and Rewards	Using technology, scheduling into the day, lifestyle
14	Active, physi, people, dementia, can, increase, health	Risk, cogniti, diseas, dementia, prevent, access, physic	Recognising physical inactivity as a factor for dementia	Other factors act as risk factors for dementia
15	walk, activ, week, feel, get, good, great	barrier, thought, present, true, pilat, husband, sore	Environment, infrastructure & lack of, motivation, Pushing through	Pushing through the barriers of physical activity

### Barriers to participation in physical activity

TMA resulted in four topics (topics 5, 6, 11 and 15) about barriers to participation in physical activity. From this, several themes were developed (Table 2).

### Work and family commitments (Topics 11 and 6)

Topics 11 and 6 are reported together as both identified being time poor, due to family and or work commitments, was a barrier for physical activity. There was a significant age trend for topics 11 and 6 (p < .001), where older people were less likely to discuss being time-poor. Males were also significantly less likely to discuss being time poor in topic 11 (p = .012). Most participants reported family commitments, busy work schedules and a lack of time as a barrier for physical activity participation. For participants who work throughout the day, lacking energy and not enough time to partake in physical activity at the end of a workday was reported, “I have an hour and a half commute to work each morning and evening. Once I get home with my two-year-old, it’s time to fix dinner for everyone, once that is done, eaten and I clean up, make sure everyone gets a bath and if I’m lucky, get an hour to play with my little girl before bedtime. I honestly don’t know when or where I can find the time for daily workouts” (F, 40 years) and “when you work 9 hours a day, I find it very difficult to exercise because you feel tired at the end of the day” (M, 35 years). Participants who worked night shifts also reported this to be a barrier to physical activity because during daylight hours, this was their opportunity to sleep, ‘Working night shift, it is difficult to exercise on these days as all you can do is work, eat and sleep’ (F, 54 years).

### Ageing, fatigue, and injury concerns (Topic 5)

Ageing, fatigue and injury concerns were expressed by participants as barriers to physical activity: ‘living with arthritis makes exercising difficult, but it is important to exercise within a manageable pain threshold’ (F, 55 years). Some crossover between barriers to physical activity and peer advice was evident throughout this theme, with many participants recognising that physical activity is an important lifestyle factor for health and used this forum as an avenue to provide support and advice to other participants, by providing suggestions on how to incorporate some form of physical activity into their lives despite experiencing fatigue, injury and at times pain “there have been some muscular skeletal setbacks, my advice is not to give up. Find what you enjoy doing, what you can manage’ (F, 58 years) and “the biggest challenge for some people regarding exercise is poor health i.e.: bad knees, aching shoulders or hips. So, no matter who are, tailer the exercise back to what you can manage, even walking for 20 minutes” (M, 61 years).

### Environmental conditions and local infrastructure (Topic 15)

Participants reported environmental conditions and local infrastructure to be a barrier. Participants indicated that they often found it more difficult to engage in physical activity in very hot or cold temperatures and reduced daylight hours “the motivation to be physically active and the soaring temperatures in Australia do not help” (F, 31 years) and “I live in the tropics and in the wet season the rain or humidity can make exercising a real challenge” (F, 52 years). Another barrier described by some participants included poor or a lack of infrastructure which made physical activity unsafe or undesirable “I think town planning makes a difference to people ability to do incidental exercise, for example where there are no footpaths, it makes it less likely that people will walk compared to when there are footpaths and bicycle path networks” (F, 54 years).

### Lack of motivation (topic 15)

A lack of motivation or finding it difficult to become motivated to perform some form of physical activity was a reported barrier, “I find the greatest impediment to being active is motivation” (F, 72 years) and “I have very little motivation and will power. I always feel great after a long walk but getting out and doing it is often a mental barrier for me” (F, 49 years).

### Enablers of participation in physical activity

Eight topics that related to enablers of physical activity (topics 1, 2, 3, 8, 9, 12, 14 and 15) were generated through the TMA process, from which several themes were developed (Table 2).

### Recognising physical inactivity as a risk factor for dementia (Topic 14)

The link between cardiovascular health, physical activity and dementia risk was recognised by participants in this research, “limiting sedentary activities, performing regular active physical activity and eating healthy diets have a significant impact on our cardiovascular health which subsequently impacts our brain health and hence reduce the occurrence of dementia” (M, 41 years). There was a strong emphasis on nutrition and diet from some of the participants, referring particularly to the Mediterranean diet: “There is some information about the Mediterranean diet and modified Mediterranean diet, with some meta-analysis from the mid-2010s” (M, 53 years). This response indicates that participants may have a greater understanding of how diet can be a modifiable risk factor for dementia risk. Other risk factors mentioned less frequently among participants in the forum included alcohol and hearing loss. Participants also expressed awareness of risk factors for dementia such as depression and social isolation as areas that physical activity can counteract. Men were significantly more likely to discuss topic 14 than women, p < .001, and there was a trend towards younger people discussing this topic (p < .001).

Participants appeared to have an understanding of the link between physical activity on overall health, including brain health and ascertained that a program such as the PD MOOC helped to increase this knowledge: “it really is interesting and gets me thinking about the positive impacts exercise can have not only with weight but with the brain too… more people need to be made aware of this fact… we really only hear about the benefits of exercise on the physical body, not so much on the brain” (F, 49years).

### Dog Ownership and deliberate incidental activity (Topic 8)

Another enabling theme that was developed included participants focusing on opportunities to integrate physical activity into their daily lives. Multiple participants reported that if they had to drive to their destination, they would deliberately park their vehicle away from the desired destination to promote physical activity: “sometimes I park my car and walk if my destination is a trekkable distance” (F, 34 years). Many responses indicated that having a dog provided a reason for participants to engage in physical activity: “I am lucky I have a dog, yep, I walk the dogs. Helps me out no end, (F, 51 years) and “I take my pug. for lots of walks along the nearby beaches” (F, 62 years). Older participants were significantly more likely to discuss this topic p < .001).

### Use of technology and rewards (Topic 13)

The feedback that technology provided participants about their physical activity was also an enabler to physical activity participation. Feedback from activity monitors or watches provided the participants with prompts and encouragement to be physically active, “Lucky I have a Fitbit watch to remind me every 30minutes to get up and walk, (F, 54 years) and “I bought a watch, the unexpected benefits of this watch is that is also checks your heart beat and lets you know when you are stressed and encourages you to do five slow breaths and when you have been inactive for one hour, encourages you to walk. It lets you know how much walking and cardio you have done each day” (M, 56years). Initiatives offered by companies through reward points was another enabling factor for physical activity participation “A friend told me about an app that allows me to earn Qantas frequent flyer points when I meet my target steps walked everyday’ (F, 56 years).

### Active Transport (Topic 12)

Participants also described ways in which they could incorporate ‘active transport’ for opportunities to engage in physical activity in their lives, such as riding their bicycle on designated bike pathways. Other opportunities for physical activity included using the stairs, “I try to use the stairs instead of the elevator” (F, 62 years). Older participants were significantly more likely to discuss the incorporation of active transport as opportunities for physical activity (topic 12, p = .02).

### Enjoyment, Organised Physical Activity, Socialisation (Topics 1 and 9)

The social aspect of participating in physical activity was a recurrent theme in this research, although our findings indicate that males were significantly less likely to discuss the relationship between enjoyment or socialisation aspects and physical activity (topic 1, p < .001). Joining a physical activity group at the local council, the gym, a leisure centre or Zumba lessons was common and often included text in the context of minimal costs to participation: ‘I have joined our over 50’s club and they have very reasonable classes, aerobic, keep fit and Zumba classes. Great fun and I get to make new friendships too’ (F, 66 years) and by ‘joining groups in your local area, is a way that older people can exercise and socialise with minimum cost’, (F, 54 years) and ‘I go twice a week to a very well-equipped gym where council has a cheaper rate and special programs for older people’ (F, 71years). The opportunity to socialise with others was an enabler to physical activity among the participants: ‘combining exercise with social activity is very helpful. I have joined a group of older women who swim almost every day. Once I started to become a part of their group, I found the experience doubly enjoyable and there is less chance of me giving up” (F, 75 years).

### Peer advice

The research generated four topics (4, 10, 13 and 15) that were directly related to peer advice (Table 2). This was in addition to participants reporting peer support as an enabler to physical activity, and to peer advice mentioned in topics focusing on barriers or enablers.

### Pushing through the barriers to physical activity (Topic 15)

Several barriers to physical activity participation were discussed in the previous section, some of which included being time-poor due to family and work commitments, lacking the energy or motivation and injury or pain. Whilst this was regularly stated in the discussion forum, participants also provided possible strategies in their responses on how they would ‘push through’ these barriers so that they could incorporate some form of physical activity into their day (time poor and no energy) .… “my busy work schedule, family commitments and lack of energy make it difficult to participate in physical activity, however, I have been trying to get up early before work to walk’ (F, 46 years), (time-poor, injury) … I work a hectic night-time schedule; at home I have four children and we run a farm. Last month I fell and hurt my knee. I went to the physio and then joined the local gym. I have made it a goal to get to the gym 3 times per week” (F, 47 years) and (lack of motivation): “there are plenty of times that I have found it very hard to motivate myself. The way I get through this is as soon as I start thinking about not doing the activity, I switch off from that thought and go into robot mode, in other words, I don’t think about it but do it. When I finish the activity, I am always feeling a lot better” (F, 53 years). Responses like this, made themes within the research cross over between barriers and enablers to for physical activity.

### Offerings of support and Advice (Topics 4 and 13)

Respondents took the opportunity to share ideas with one another around engaging in physical activity and provided offerings of support and advice. These offerings of support and advice were frequent enough that an entire topic was generated through the TMA process: “being outside in the environment, exercising with a friend where you can talk and laugh, they are free and there are good online workouts. Small modifications to daily routines e.g., getting off the bus one stop earlier and walking a bit further, walking upstairs instead of using the lift” (F, 58 years). Other advice spoke about tailoring physical activity to your current levels of fitness and needs: “asking for a good physiotherapist, you would get a series of exercises that are tailored to your needs, state of health and capabilities. Most of the exercises can be modified to suit” (F, 54 years). For other members of the forum, advice on initiating and maintaining physical activity was provided: “the hardest part is beginning and actually getting yourself into the gym or going for that walk. Once you start and maintain a regular routine, it becomes easier” (M, 23 years). Participants provided specific examples around increasing physical activity across the age span. For example, “I like swimming. It is a good activity suitable for people of different ages” (F, 32 years).

### Self-discipline and establishing a routine (Topic 10)

Throughout the responses, participants indicated that the challenge was initiating physical activity, particularly after a lengthy hiatus. Participants encouraged others in the discussion forum that self-discipline and adherence to their physical activity was important so that a routine can be established, ‘For me it is a matter of self-discipline. I was always able to find an excuse not to exercise, until 6 months ago, when I joined the local gym and found a trainer. I now attend weekly, have both a physical and aerobic program, and feel better overall’ (F, 72 years and ‘I agree with you- rain or shine we are out for a minimum of 30 min/day’ (F, 67 years).

## Discussion

The cohort’s responses to the research questions ‘Are there things that make it difficult for you to participate in more physical activity’? and ‘If you increased your physical activity levels, what tips could you give others to do the same’, yielded replies from which fourteen themes were identified. Our research expands on previous enabler and barrier research to physical activity by examining the reasons that facilitate and limit individuals’ engagement with physical activity, in particular those individuals who have an interest in dementia risk reduction, and this provides new and exciting insights from a novel, online platform to encourage others to engage in physical activity via peer support and peer advice.

This research identified barriers to physical activity such as time constraints, fatigue and injury concerns, lack of self-discipline, environmental conditions, and low motivation. Additionally, participants reported that physical activity is beneficial to brain health and provided an opportunity for socialisation by joining a planned physical activity group such as a gym or local council group. Dog ownership and the use of technology such as smart watches also enacted as enablers for physical activity in this research. These barriers and enablers have been reported in previous literature (15, 17, 30–36). It is worth noting that the similarities yielded by the responses from this online discussion forum suggests that this method of qualitative data collection provides an alternative to traditional face to face interviews and therefore would appear to be a trustworthy data collection method for qualitative research. Participation in this research involved no sampling criteria, no restriction on age, gender, socio-economic status, employment and/or education. Similarly reported in online programs and stroke by (14), this online discussion forum encouraged participants, from different countries and diverse demographics to post on each conversation thread, whilst permitting participants to feel less inhibited which allowed them to express honest opinions about potentially sensitive issues. Advantages of using this online forum method for data collection also allows participants to communicate their opinions and capture participant perspectives from a wide geographical area with anonymity. Taking advantage of a well-established online forum such as the PD MOOC offers an opportunity for cross communication and shared support among participants, where a personalised support system and trust is developed (14). Further, the cross communication between participants can strengthen the understanding of the benefits of physical activity for dementia risk reduction and brain health and add depth to the themes in qualitative research (14).

The findings from this research shines a new light on the potential impact of peer support and advice, given the paucity of literature that investigates peer support and peer advice from online participants that share and describe the benefits of physical activity as a modifiable risk factor for dementia. To our knowledge, this mode of peer support and advice among PD MOOC participants is unique in that it provides an understanding from participants, from various backgrounds, demographics and levels of knowledge relating to physical activity, dementia and/or both, concerning ways to support one another to become more physically active and reduce their dementia risk. This is based on an understanding that what the public know about physical activity in terms of a risk reduction strategy for dementia is critical to formulating health strategies and campaigns (10).

Our research suggests that large online discussion forums such as the PD MOOC are an effective method to deliver, seek and provide support to many. Participants with an interest in decreasing their dementia risk can if enabled provide an online peer support forum and can act as an avenue for information among all populations to obtain dementia knowledge associated with physical activity and to increase the rates and motivational factors of others to perform physical activity. Research conducted by (37) explored the state of knowledge regarding what young Australian’s know of modifiable factors for dementia risk reduction. They found that young adults had the greatest potential to change their dementia risk and reported 70% of their participants had a limited understanding of dementia and 20% of their participants reported that they believed that there were no modifiable risk factors for dementia, including physical activity. Knowledge of dementia risk factors such as physical inactivity needs to be translated into the wider public as a health strategy campaign. Online platforms have the potential to become an integral health information strategy, where more e-health programs can be provided with a citizen-centric system of care, increasing social inclusion and people empowerment (38).

In our research, social support was a strong enabling theme among the participants with many posts encouraging physical activity participation by joining local clubs or activity groups or by buddying up with a friend to encourage one another to adhere and motivate one another to be physically active. Social support for physical activity has the potential to motivate individuals to initiate physical activity (39). Social support has been consistently reported as a significant important factor associated with physical activity as it provides confidence. The relationship between walking and strong social networks maybe cause-effect, as individuals who are out walking have the potential to meet and socialise with others more than those who remain at home. Others have found that scheduling activities is a core component of effective care management for dementia (40). Such scheduling including diarising and scheduling physical activity into the day by buddying up with someone to perform physical activity; it is believed to improve accountability to oneself and the buddy. It would seem therefore that online peer advice and peer support around physical activity could play a similar role, and this might be an important area for future investigation.

For this cohort, cost of physical activity was not a theme that repeatedly imposed a limitation to physical activity. Perhaps this was attributed to the time of the forum discussion where COVID_19 had placed restrictions on attending formalised physical activity programs such as personal training groups and gymnasiums. Instead, participants had to rely on other independent forms of physical activity participation such as walking with family members or COVID buddies independently. The impact of cost to physical activity is an important area that could be explored in the future in an online forum discussion.

### Strengths and Limitations

The current research was undertaken using a large online forum which offered a peer supported environment. In line with the advantages reported by (14), the online approach allowed participants to take part in this research at their own convenience (time and place). It is noted that whilst participants did not require any qualifications or background knowledge of dementia to complete the course, making the PD_MOOC open to everyone and readily accessible to all, a level of effort and endurance is required to complete the course in its entirety.

An additional advantage of using an online forum is the potential for international sampling. Although limited by a requirement to be fluent in the English language, future iterations might overcome this limitation with the implementation of online tools and the development of the PD MOOC in diverse languages to cater for Culturally and Linguistically Diverse populations.

Despite the enforced restrictions of the recent COVID-19 pandemic, this online approach holds potential to allow qualitative research to continue, as it provides another method to collect data without the need to attend face-to-face interviews or focus groups (41). During the COVID-19 pandemic, research conducted via the MOOC platform allowed for an alternative recruitment method that adhered to physical distancing regulations or recommendations. It is possible the use of topic modelling analysis may have limited the visibility of less frequent topics, and exemplars of each topic are not ever fully representative of the topics they are weighted most heavily on. However, this approach enabled a sample size larger than one that could have been recruited in interview/focus group style research, allowing for the expression of a broad range and depth of views. A further strength was that the researchers were unlikely to have influenced participants’ responses as they were unknown to participants. This may have encouraged more thoughtful and in-depth responses to the questions posed on the discussion forum. Posts enabled a collaborative approach by participants for identifying barriers and enablers and provided suggestions and co-created solutions to physical inactivity. Despite the inability to ask clarification questions or to probe forum participants, they could read and reply to each other’s posts in an asynchronous way, with online discussions allowing an in-depth exploration of themes about barriers and enablers.

The research included participants who were interested in physical activity and dementia, highly educated, older than population averages, predominantly female, and willing to take part in an online discussion board; therefore, these findings may not be representative of the general ‘population. The size and potential impact of this bias is a consideration for future research using the PD MOOC or similar platforms.

## Conclusion

Using online discussion forums allowed for peer gathered information which is important as it promoted person to person affirmations and encouragements rather than prescriptive forms of physical activity behaviour advice for people interested in increasing their physically activity to reduce their risk of developing dementia.

Current recommendations for physical activity participation reflect a strong evidence base supporting the multiple benefits of physical activity for preventative, therapeutic, maintenance and improvement in physical function and more recently dementia risk reduction. The novel method adopted in this research involving online discussion forum captured ideas from a diverse group of participants sharing strategies for increasing physical activity. Future research may benefit from further examining the role of peer advice in dementia risk reduction initiatives.
